# Systems of conductive skin for power transfer in clinical applications

**DOI:** 10.1007/s00249-021-01568-8

**Published:** 2021-09-03

**Authors:** Andreas P. Kourouklis, Julius Kaemmel, Xi Wu, Evgenij Potapov, Nikola Cesarovic, Aldo Ferrari, Christoph Starck, Volkmar Falk, Edoardo Mazza

**Affiliations:** 1 Department of Mechanical and Process Engineering, Institute for Mechanical Systems, ETH Zurich, Leonhardstrasse 21, 8092 Zurich, Switzerland; 2 EMPA, Swiss Federal Laboratories for Material Science and Technology, Überlandstrasse 129, 8600 Dübendorf, Switzerland; 3 Department of Cardiothoracic and Vascular Surgery, German Heart Center Berlin, Augustenburger Platz 1, 13353 Berlin, Germany; 4 Department of Health Sciences and Technology, ETH Zurich, 8093 Zurich, Switzerland

**Keywords:** Left ventricular assist devices (LVAD), Cardiac implantable electronic device (CIED), Driveline infections (DLIs), Foreign body reaction (FBR), Surface topography, Biofilm

## Abstract

The primary aim of this article is to review the clinical challenges related to the supply of power in implanted left ventricular assist devices (LVADs) by means of transcutaneous drivelines. In effect of that, we present the preventive measures and post-operative protocols that are regularly employed to address the leading problem of driveline infections. Due to the lack of reliable wireless solutions for power transfer in LVADs, the development of new driveline configurations remains at the forefront of different strategies that aim to power LVADs in a less destructive manner. To this end, skin damage and breach formation around transcutaneous LVAD drivelines represent key challenges before improving the current standard of care. For this reason, we assess recent strategies on the surface functionalization of LVAD drivelines, which aim to limit the incidence of driveline infection by directing the responses of the skin tissue. Moreover, we propose a class of power transfer systems that could leverage the ability of skin tissue to effectively heal short diameter wounds. In this direction, we employed a novel method to generate thin conductive wires of controllable surface topography with the potential to minimize skin disruption and eliminate the problem of driveline infections. Our initial results suggest the viability of the small diameter wires for the investigation of new power transfer systems for LVADs. Overall, this review uniquely compiles a diverse number of topics with the aim to instigate new research ventures on the design of power transfer systems for IMDs, and specifically LVADs.

## Introduction

**Fig. 1:**
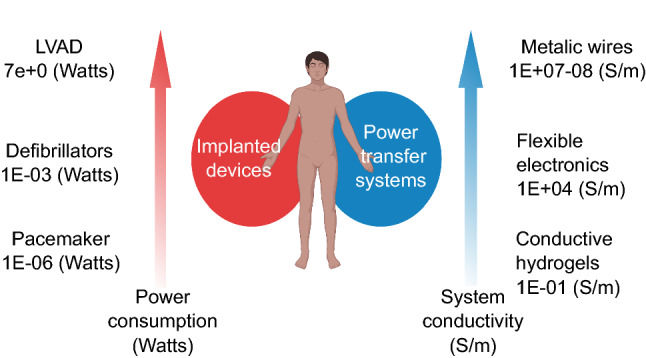
Different IMDs and power transfer systems combined for the therapeutic benefit of human patients. Increased transparency of the red arrow reflects IMDs with lower power consumption. On the right side, selected power transfer systems to support the function of IMDs. Increased transparency of the blue arrow corresponds to power transfer systems with lower electrical conductivity. Currently, drivelines composed of insulated metallic wires are the only conductive system which is compatible with LVADs

The emergence of digital technology catalyzes a generation of medical breakthroughs and offers unprecedent access to the management of human disease (Topol 2019). Along this line, the widespread use of digital hardware, such as smart phones and wearable sensors, has inspired significant innovations on the design of novel diagnostic solutions (Chandrasekhar et al. 2018; Yang et al. 2020). On a similar track, harnessing the strengths of cutting-edge technologies has the potential to benefit thousands of patients receiving implanted medical devices (IMDs) with therapeutic electromechanical function, such as cardiac implantable electronic devices (CIEDs) and left ventricular assist devices (LVADs). The latter type of medical intervention poses a non-trivial challenge which primarily pertains to the development of suitable power transfer systems for supporting the requirements of different IMDs, Fig. 1. Comparing commonly used IMDs in patients with cardiac problems, LVADs consume significantly higher power (~ 7 W) than defibrillators (10^–3^ W) and pacemakers (10^–6^ W) (Ben Amar et al. 2015). For this reason, the use of well-insulated transcutaneous metallic wires (e.g., drivelines) is the only available commercial solution that is currently applied with LVADs. On the contrary, wireless (e.g., flexible electronics: ~ 10^4^ S/m) and less invasive solutions (e.g., conductive hydrogels: ~ 10^–1^ S/m) for power transfer are not sufficient to support LVADs due to the significantly lower electrical conductivity compared to metallic wires (~ 10^7^–10^8^ S/m) (Kim et al. 2011; Sirivisoot et al. 2014*)*.

LVADs, which are on the spotlight of this review, expose patients to the risk of infection and foreign body reaction as part of short- and long-term pathological responses, respectively (Zinoviev et al. 2020). A leading cause of infections in LVADs is the use of percutaneous LVAD drivelines to convey power and information through the human body. Although wireless solutions of power transfer may be resolutive, their clinical implementation faces technical challenges and is still far from becoming part of the mainstream clinical practice. Therefore, power drivelines remain the most reliable strategy to cover the power requirements of LVADs, urging the need to generate new driveline systems with improved biological compatibility and optimal power characteristics.

Here, motivated by the cause-to-effect relationship between percutaneous LVAD drivelines and the risk of infections, we present different in vitro and in vivo models of the skin that may be used to identify critical factors for the progression of infections. Furthermore, we review a number of alternative technological solutions for the effective and less destructive transfer of power in IMDs. Finally, we propose the creation of conductive skin systems with high-conductivity components as an innovative solution towards the risk-free and long-term integration of power and signal drivelines connected to IMDs. Altogether, this essay provides a critical perspective on the current challenges and opportunities associated with power transfer in the human body, with the objective of drawing new directions on the design of biologically compatible and infection-free IMDs, such as LVADs.

## Clinical challenges associated with the power transfer of LVADs

LVADs are implanted in adult patients with heart failure (HF) to restore blood perfusion back to physiological levels. About half of the LVADs implanted nowadays serve as destination therapy (Kirklin et al. 2017), while the rest address the following treatment strategies: bridge to transplantation, bridge to candidacy and bridge to recovery (Ponikowski et al. 2016). Together with the increasing number of patients with end-stage HF and the shortage of donors for organ transplantation, the advancements in LVAD technology led to the preferential use of LVADs in the form of destination therapy. Current state of the art LVADs include an implanted pump connected to an external battery and a controller via a well-insulated percutaneous driveline (Schmid Daners et al. 2017). However, LVADs can also become subject to major complications, including driveline-specific infections (DLIs) (Hannan et al. 2019). Relevant epidemiological studies reveal at least one incidence of DLI in the first year of LVAD support for 19% of the patients, while the peak of incidence occurs at approximately 6 months after implantation (Goldstein et al. 2012; Pavlovic et al. 2019).

DLIs are associated with the full skin injury and the ongoing percutaneous presence of LVAD drivelines that together impair the process of wound healing. Skin is composed of several layers with distinct contributions into the process of wound healing (Kwon et al. 2018; Rodrigues et al. 2019). The epidermis contains a stratified epithelium that mediates barrier function against external environmental factors. Anchored to that, the fibroblast-rich dermis has a substantial role into the mechanical properties of the skin tissue. Wound healing is a multi-step process that involves blood clot formation, inflammation, re-epithelialization, tissue granulation, neovascularization, and tissue contraction (Rousselle et al. 2019; Shaw and Martin 2009). During re-epithelialization, keratinocytes migrate towards the injured tissue to re-establish a new epithelium (Bamberger et al. 2005). However, the implanted LVAD drivelines occupy the center of the wound, altering the biomechanical and biochemical features of the injured site in a manner that burdens keratinocyte migration from the margins to the center of the scission (Pensalfini et al. 2018; Wahlsten et al. 2019; Wietecha et al. 2020).

In addition to that, previous studies suggest a no-slip condition between keratinocytes and LVAD drivelines, which promotes distal epidermal growth and subsequently sulcus formation around the drivelines (Großhauser et al. 2015). In effect of that, the weak sealing between epidermis and drivelines gives rise to a durable breach for biofilm formation of fungal and microbial composition, which can further migrate into the skin causing  DLI (Qu et al. 2020). Yet,  how the intercellular crosstalk between keratinocytes and dermal fibroblasts affects wound healing in the presence of percutaneous drivelines remains unclear. In this direction, forthcoming studies with systematic control on driveline motion, surgical details, and patient health will be critical to investigate the underlying cellular interactions, identifying new therapeutic targets against DLIs (Dean et al. 2015; Zierer et al. 2007).

## Clinical protocols against driveline infections (DLIs)

**Fig. 2:**
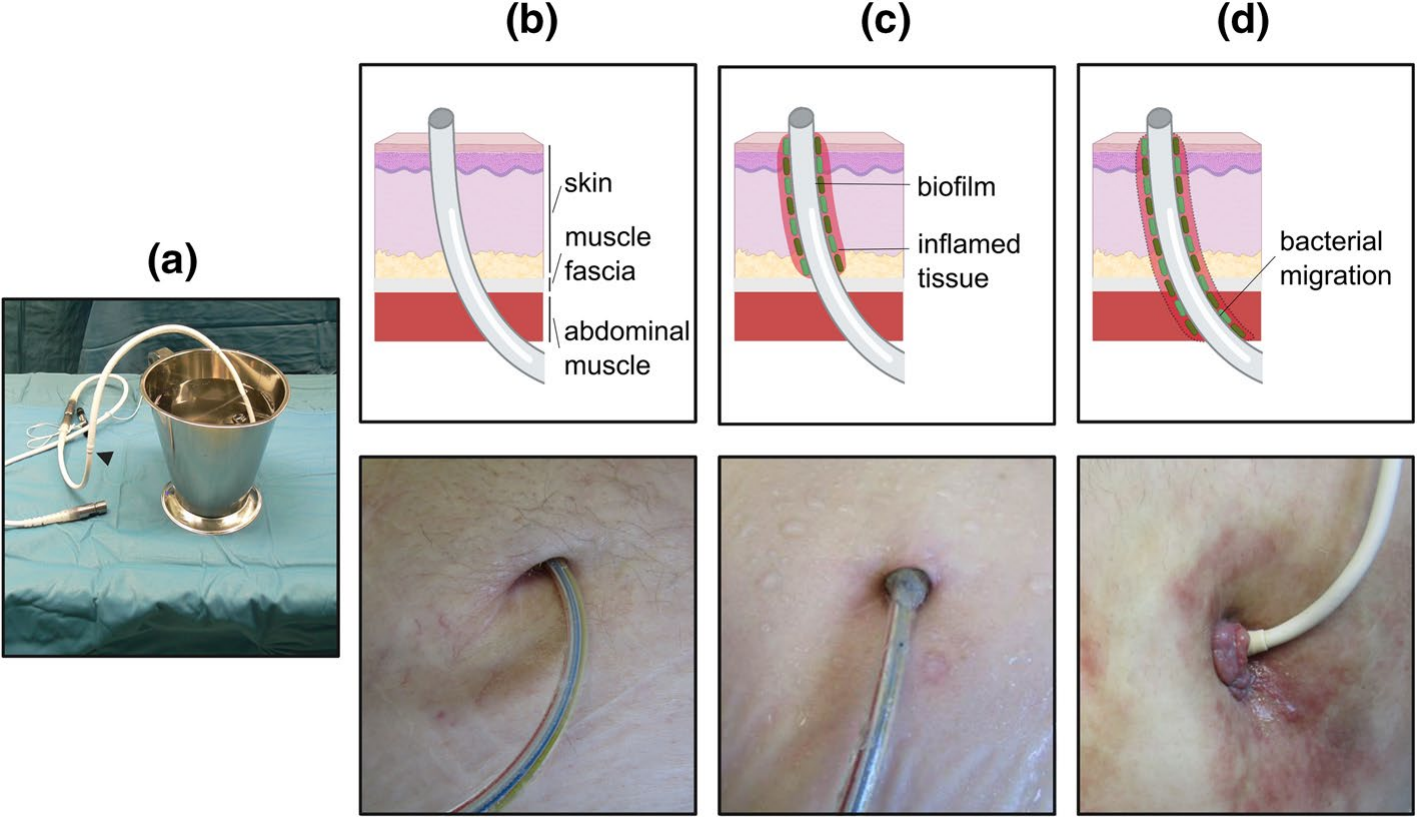
Percutaneous LVAD drivelines may be subject to infections. (**a**) Pre-implanted LVAD driveline with the velour section immersed in the container. (**b**) Implanted LVAD driveline through the human skin. The lower image depicts an infection-free exit of the percutaneous LVAD driveline. (**c**) Biofilm formation at the upper layers of the human skin may lead to superficial infection sparing the muscle fascia. The lower image depicts a patient with superficial infection. (**d**) Bacterial migration into the lower layers of the skin may lead to deep infection involving the muscle fascia. The lower picture depicts a patient with deep driveline infection. The cartoons of this review were created with the assistance of Biorender.com

In the clinical context, LVAD infections are distinguished in three different groups: LVAD-specific, LVAD-related and non-LVAD-related infections. The DLIs, which is a top interest of this review, together with infections of the adjacent tissue belong to the category of LVAD-specific infections. The progress and the severity of DLIs are correlated with the anatomical position of the affected tissue compartments. Specifically, depending on whether the muscle fascia or the deeper muscle tissue is involved (Fig. 2), the DLIs are further split into superficial and deep DLIs, respectively (Hannan et al. 2011). The diagnosis of LVAD-specific infections, and particularly superficial DLIs, is based on the detection of several clinical symptoms (erythema, purulent discharge, and increased temperature) together with microbiological, echocardiographic and computed tomography studies (Hannan et al. 2011). Moreover, clinical examinations of white blood cells scintigraphy (de Vaugelade et al. 2019), positron emission tomography-computed tomography (de Vaugelade et al. 2019; Ten Hove et al. 2021) and the combination of fluorescence in situ hybridization and polymerase chain reaction (Schoenrath et al. 2020) ascertain additional diagnostic power to distinguish between superficial and deep infections.

### Post-operative protocols

The majority of DLIs coincide with the presence of Gram-positive and Gram-negative bacteria like *Staphylococcus aureus* and *Staphylococcus epidermidis*, while the strain of *Pseudomonas aeruginosa* leads the Gram-negative category. Even rarely, fungal, viral and protozoal pathogens may also trigger DLIs with life threatening effects (Hannan et al. 2019; Qu et al. 2021; Zinoviev et al. 2020; Maly et al. 2014). The standard treatment against DLIs combines anti-infective therapeutic protocols with specialized procedures of wound therapy and surgical intervention. A challenging factor against the elimination of bacterial pathogenesis comes from biofilm formation at the interface of drivelines with the injured skin (Fig. 2b). Correct evaluation of the biofilm’s age is critical for the selection of anti-infective agents that can either eradicate or suppress pathogen distribution (Trampuz et al. 2020).

Another possible route to tackle severe DLIs is the treatment by means of surgical interventions, such as driveline relocation with wound debridement (Kusne et al. 2017; Pieri et al. 2016; Trampuz et al. 2020). Equally critical is the practice of meticulous wound care protocols that are well adjusted to the needs of the different stages of DLIs. The progression of DLIs and the characteristics of percutaneous wounds are strong determinants of the selected therapeutic protocol, that can involve wound dressing in combination with supplementary treatments, such as negative pressure vacuum-assisted closure therapy, cold atmospheric plasma, and antibiotic beads (Bernhardt et al. 2020; Haddad et al. 2020; Hilker et al. 2017; Kilo et al. 2020; Sezai et al. 2020). In many cases however, the therapeutic benefits are decapacitated by relapsing infections related to biofilm persistence. Against this adversity, additional therapeutic support may include bacteriophage treatment, long-term suppression antibiotic therapy, and as a last option device exchange (Kusne et al. 2017; Mulzer et al. 2020).

### Preventive measures

To avoid the post-operative escalation of DLIs, the clinical procedures have also adopted an array of preventive measures. A key element for the prevention of LVAD infections is the administration of perioperative anti-microbial prophylaxis, similar to other types of cardiac surgery (Kusne et al. 2017). This treatment is further combined with surgical protocols, such as the double tunneling method to inhibit ascending DLIs (Fleissner et al. 2013; Wert et al. 2018). Other strategies focus on trauma prevention and its impact on the risk of infection. To this end, surgical sutures are regularly applied to stabilize drivelines at the exit site, limiting the extend of injury from potential mechanical motions (Kusne et al. 2017).

On the sidelines, the management of post-operative complications, including acute infections, is starting to be largely recognized and has led to the introduction of protective envelopes which support the controlled release of antibiotics for at least 1 week after deployment (Biffi 2019). Although this strategy has not been tested in LVAD protocols, the use of drug-eluting polymeric envelope around CIEDs in a large clinical trial was proven more effective compared to simple flushing of the generator pocket and ensuing systemic treatment (Krahn et al. 2018; Tarakji et al. 2019). Based on these results, novel strategies for the use of antibiotic envelopes in combination with neurostimulators (e.g., deep brain, sacral nerve, vagus nerve stimulators) and LVADs shall be subject to further studies to gain indications relevant to driveline protection.

## Skin models for the study of driveline infections

### In vitro models of human skin

The percutaneous implantation of LVAD drivelines comes together with a series of mechanical and chemical stressors that can facilitate the progression of DLIs. However, the mechanisms that drive cellular responses around the drivelines elude our understanding due to the shortage of available patients for exploratory mechanistic studies. To this end, in vitro systems that recapitulate critical conditions of skin physiology may be used to uncover the mechanisms downstream of driveline-generated stressors. A large number of in vitro skin models are built through a bottom-up approach (Randall et al. 2018), where an initially formed dermal scaffold supports the organization of an upper epidermal layer. This artificial epidermis is regularly comprised of keratinocyte cells, attaining a platform to investigate various mechanisms, such as wound healing and regeneration (Deshayes et al. 2018). Similar in vitro systems can also host studies for the evaluation of new biocompatible and infection-resistant percutaneous devices (Bolle et al. 2020a, b) or to simply uncover the individual effects of different bacterial strains (Jahanshahi et al. 2020; Koval et al. 2019; Popov et al. 2014; Zinoviev et al. 2020). In spite of this potential, the majority of in vitro skin models are deprived of an efficient circulatory network, hindering its exposure to biologically relevant immune responses (Kim et al. 2019; Miyazaki et al. 2019; Saleh and Bryant 2017). In contrast, natural skin explants of human and animal origin sustain an organotypic complexity which is associated with pathological responses, such as foreign body reaction (FBR) and infection (Dellambra et al. 2019; Griffin et al. 2020; Maboni et al. 2017; Rakita et al. 2020; Schaudinn et al. 2017; Torres et al. 2020; Yoon et al. 2019). Future development in this direction shall include the implantation of percutaneous drivelines in perfusable skin explants to further elucidate the dynamic biological signals that instruct DLIs (Moniz et al. 2020; Ternullo et al. 2017).

### Animal models

In vivo models have been largely used to capture the progression of DLIs and FBR in pre-clinical studies (Dondossola et al. 2016; Greenfeld et al. 1995; Isenhath et al. 2007; Jarvik et al. 1998; Toba et al. 2011; von Bayern et al. 2008; Zierer et al. 2007). The selection of a particular animal model is influenced by several factors, such as the driveline system, the conditions of animal handling, the investigated bacterial strain, and the desired standards for physiological resemblance to the human skin (Carney et al. 2009; von Bayern et al. 2008). In this case, large animals are usually preferred for the exploration of promising LVAD technologies, especially due to adequate surgical space in favor of animal comfort and surgical precision (Carney et al. 2009; Kitao et al. 2011; McGee et al. 2014; Monreal et al. 2014; Tuzun et al. 2007; Weiss et al. 2012). Among larger animals, the physiology of the porcine skin closely resembles that of human (Ashara and Shah 2016; Grada et al. 2018; Tsai et al. 2019; Vodička et al. 2005). Yet, many driveline studies employ goats or sheep that enable driveline stabilization onto a minimally perturbing dorsal site which is further protected by specialized protective vests and amenable breeding conditions (Carney et al. 2009; Großhauser et al. 2015; Lee et al. 2013). In conclusion, the selection of different skin models is well correlated with the leading pre-clinical questions and the most recent standards for animal welfare. In this direction, the rigorous pre-clinical assessment of DLIs shall put together a combinatorial strategy to harness the distinct advantages of more than one category of the aforementioned skin models.

## Foreign body reaction, lessons from the past

A critical condition for the successful incorporation of IMDs in a host organism is related with their capacity to minimize FBR. This is a common biological challenge that escalates to the assembly of fibrotic capsules around artificial materials comprising IMDs (Anderson et al. 2008). Fibrotic capsules resemble granulation tissues with immature vascularization, which may eventually impede the electromechanical performance of IMDs. For instance, capsule formation around CIEDs attenuate signal transmission in the cardiac tissue, leading to increased power consumption or even local overheating (Dvorak et al. 2012; Li et al. 2020). To combat this problem, previous studies modified implanted devices with different grades of biochemical molecules (Liu et al. 2008; Park et al. 2019; Weigel et al. 2018). Consistent to that, novel biomaterials enable the design of device sleaves that prevent FBR (Davenport Huyer et al. 2020; Robotti et al. 2020), alleviating the mismatch between implants and the tissue microenvironment.

Moreover, the size, microarchitecture, and mechanical properties of implanted objects constitute additional design parameters to mediate the extend of FBR (Helton et al. 2011). For instance, implants of smaller size and lower elastic modulus demonstrate significantly less FBR (Sanders et al. 2002). Likewise, textured surfaces have been shown to weaken FBR, as validated by the formation of thinner and less dense fibrotic capsules (Johansson et al. 2009; Picha and Drake 1996; Ward et al. 2002). These findings underline a mechanistic correlation between the material properties of the implanted objects and the extent of FBR, that shall be harnessed to design new power drivelines with only benign FBR.

## Alternative solutions for high-power transfer

### Wireless power transfer

**Fig. 3:**
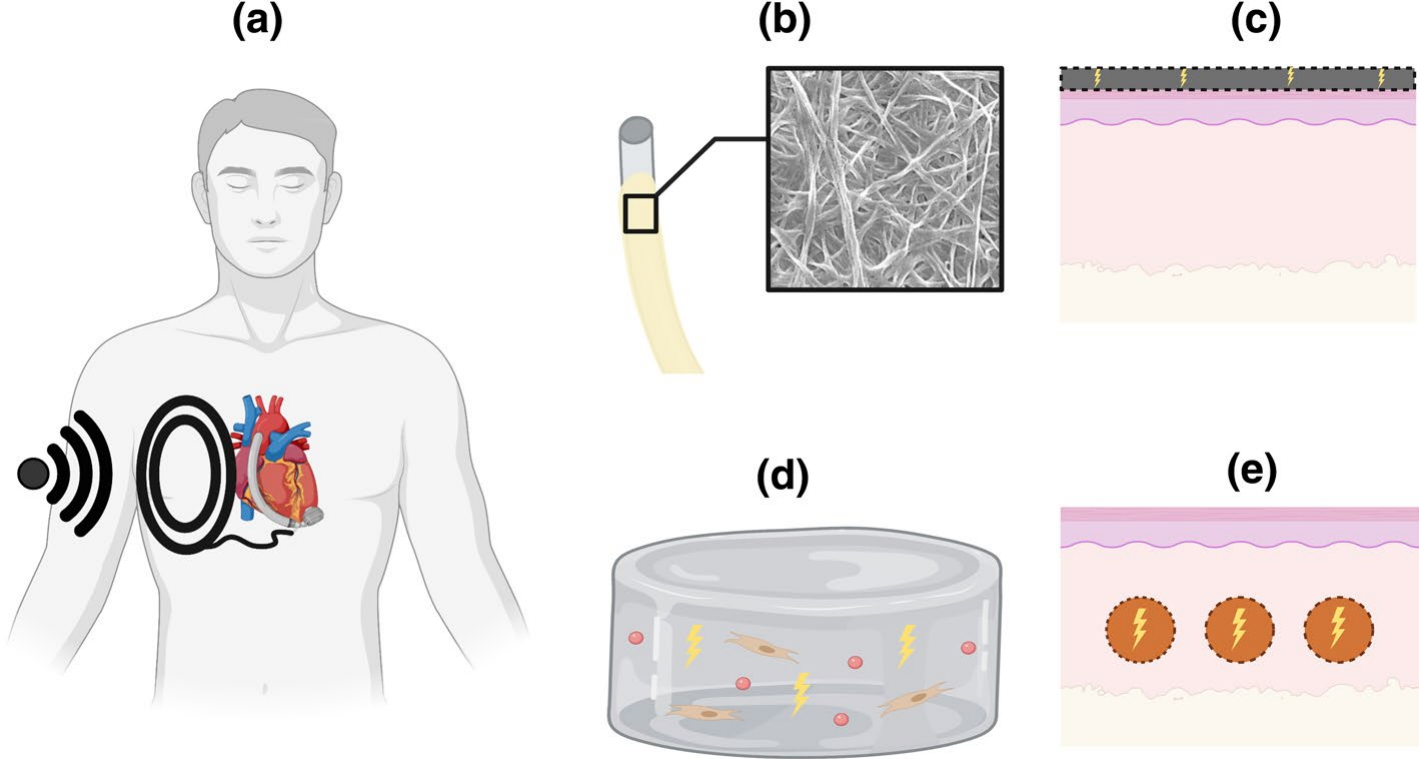
New solutions for power transfer into the human body with the aim to eliminate the problem of infection. (**a**) TETS, (**b**) driveline modification with physical and biological features for enhanced antibacterial properties and integration with skin [inner image depicts anti-fibrotic biosynthesized cellulose (Robotti et al. 2020)], (**c**) laminated flexible electronics on epidermal tissue, (**d**) conductive biological hydrogels with controlled cellular composition and conductive particles, and (**e**) new systems of conductive skin accommodating highly conducting metallic wires in 3D skin substitutes. The cartoons were created with Biorender.com

Wireless power transfer has the potential to solve the problem of DLIs. With this ambitious goal, the transcutaneous energy transfer systems (TETS) are widely investigated with the aim of leading to fully implanted and infection-free LVADs (Fig. 3a). In one of the first cases that combined TETS in LVAD implantation, the transfer of power was carried out by induction coupling between an external and an implanted coil, after the contactless transmission of direct current through the physiological barrier of the human skin (Leuck 2015; Mehta et al. 2001). Still, formidable technical challenges render wireless solutions unsuitable for the hazard-free support of LVADs.

TETS intolerance to coil misalignment burdens their systematic application to a patient population with varying body types (El-Banayosy et al. 2003; Waters et al. 2014). To tackle this challenge, recent efforts developed free-range resonant electrical energy delivery (FREE-D) systems that employ magnetic resonance to successfully transmit power in both short- and long-range distances (Waters et al. 2018). In a small-scale clinical trial, the use of coplanar energy transfer (CET) proved to be feasible for powering continuous flow LVADs (Pya et al. 2019). Despite the overall progress, the implementation of a wireless-based solution for power transfer in LVADs is far from becoming part of the clinical routine. In this direction, new wireless solutions against tissue overheating and fibrotic responses will be essential to avoid power outage and tissue damage with life threatening consequences (Letzen et al. 2018).

### Physical and biological functionalization of power drivelines

The effects of percutaneous drivelines on the skin tissue may well reflect the bi-directional communication between skin cells and the material properties of LVAD drivelines. This hypothesis is supported by an inherent cellular mechanism which integrates mechanical and biochemical cues of the extracellular environment into downstream signals of tissue repair (Tschumperlin et al. 2018). Up to date, biomaterials with controlled chemistry, stiffness, shape, and surface topography have been successfully formulated to coordinate cellular responses in wound healing and tissue regeneration (D’Ovidio et al. 2019; Guimarães et al. 2020; Kaylan et al. 2017; Kourouklis et al. 2014; Li et al. 2017; Murray et al. 2019; Ragelle et al. 2018; Rahmati et al. 2020; Tylek et al. 2020). In the same context, the material properties of the drivelines may interfere with the cellular functions in the proximal skin tissue. Thus, the design of drivelines with controlled presentation of biochemical and biophysical cues (Fig. 3b) offers an alternative strategy for instructing tissue responses that reduce the chance of DLI.

Previous clinical studies showed that the smooth silicone jacket of HeartMate II drivelines attaches more firmly onto the wound compared to porous velour (McCandless et al. 2015), suggesting that the physical characteristics of LVAD drivelines can influence their interaction with injured skin. In contrast, the incorporation of porous features on the surface of exploratory percutaneous devices corresponded to enhanced dermal and epidermal incorporation without evidence of infection in mouse studies (Fukano et al. 2010). Interestingly, mock drivelines modified with a fibrous surface demonstrated strengthened adhesion with human skin equivalents (HSE) (Bolle et al. 2020a). For the same in vitro set up, however, the percutaneous implantation of the fibrous mock drivelines into HSE did not inhibit downward epidermal growth (marsupialization) which is a histological condition connected with infected percutaneous LVAD drivelines. Apart from the surface features, the size and stiffness of the drivelines may also affect the progression of DLIs. In a relevant clinical study, the thinner and more compliant drivelines, as indicated by the force load (N) required to push a gauge to a certain distance (5 mm) against the drivelines, restricted complications related to percutaneous implantation (Imamura et al. 2017). Specifically, HeartMate II drivelines (Diameter (*D*) ~ 6 mm, 3 N) induced fewer cases of DLIs compared to the larger and stiffer drivelines of DuraHeart (*D* ~ 9 mm, 40 N) and EVAHEART (*D* ~ 9.5 mm, 50 N).

In a similar context, the biological functionalization of drivelines aims to eradicate DLIs through increased biological compliance. This goal may  be achieved by attaching extracellular and cellular components on the surface of the drivelines, recapitulating critical features of skin architecture (Debels et al. 2015; Sheikholeslam et al. 2018). For instance, the biochemical functionalization of 2-dimensional (2D) silicon and velour substrates with type I collagen is shown to reduce non-specific protein adsorption and enhance fibroblast adhesion (Hussain et al. 2016), indicating potential benefits from the dermal functionalization of LVAD drivelines. Similarly, fibroblast seeding on top of 3-dimensional (3D) mock drivelines promoted upward epidermal growth, inhibiting bacterial migration in HSE (Bolle et al. 2020a, b). Overall, the strategy of driveline functionalization can exploit previously recorded cell-material relationships to expedite the design of power transfer systems with enhanced biocompatibility and infectious-resistant effects.

### Engineering new systems of conductive skin

A significant stepping-stone towards the next generation of power transfer systems will be the design of highly conductive elements within scaffolds of increased biological mimicry. Yet, these devices will not only incur the high risk of infection and FBR, but that of the electrical hazard against fragile biological parts as well. Currently developed conductive systems bear properties that partially protect them against similar problems. For instance, although flexible material systems, the so called “electronic skin”, may serve as well-laminated power transfer systems (e.g., coil, solar panels, piezoelectric generators) onto the human skin (e-skin, Fig. 3c), the uncertain biological compatibility and low-power characteristics (μWatts–mWatts) restrain their use in LVAD applications (García Núñez et al. 2019; Hammock et al. 2013; Kim et al. 2011; Li et al. 2016; Zhu et al. 2020).

A different approach constitutes from composite systems that blend high-conductivity constituents with biopolymers to engineer tissue scaffolds with improved electrical properties (Fig.3d) (Guo and Ma 2018; Min et al. 2018; Walker et al. 2019). Following this strategy, the addition of carbon nanotubes and polymer nanofibers in cellular scaffolds has been shown to increase conductivity without disrupting cellular viability (MacDonald et al. 2008; Sirivisoot et al. 2014). Similarly, electrode embedment in conductive biological scaffolds enhanced both the electrical transmission and the anti-fibrotic responses (Cheong et al. 2014). However, in spite of the apparent biological affinity, the achieved conductivity (~10^−1^ S/m) remains significantly lower than that of metallic wires (10^7^–10^8^ S/m) (Sirivisoot et al. 2014), dismissing the use of conductive hydrogels as power transfer systems in LVAD drivelines.

### Conductive wires with controlled physical features

**Fig. 4:**
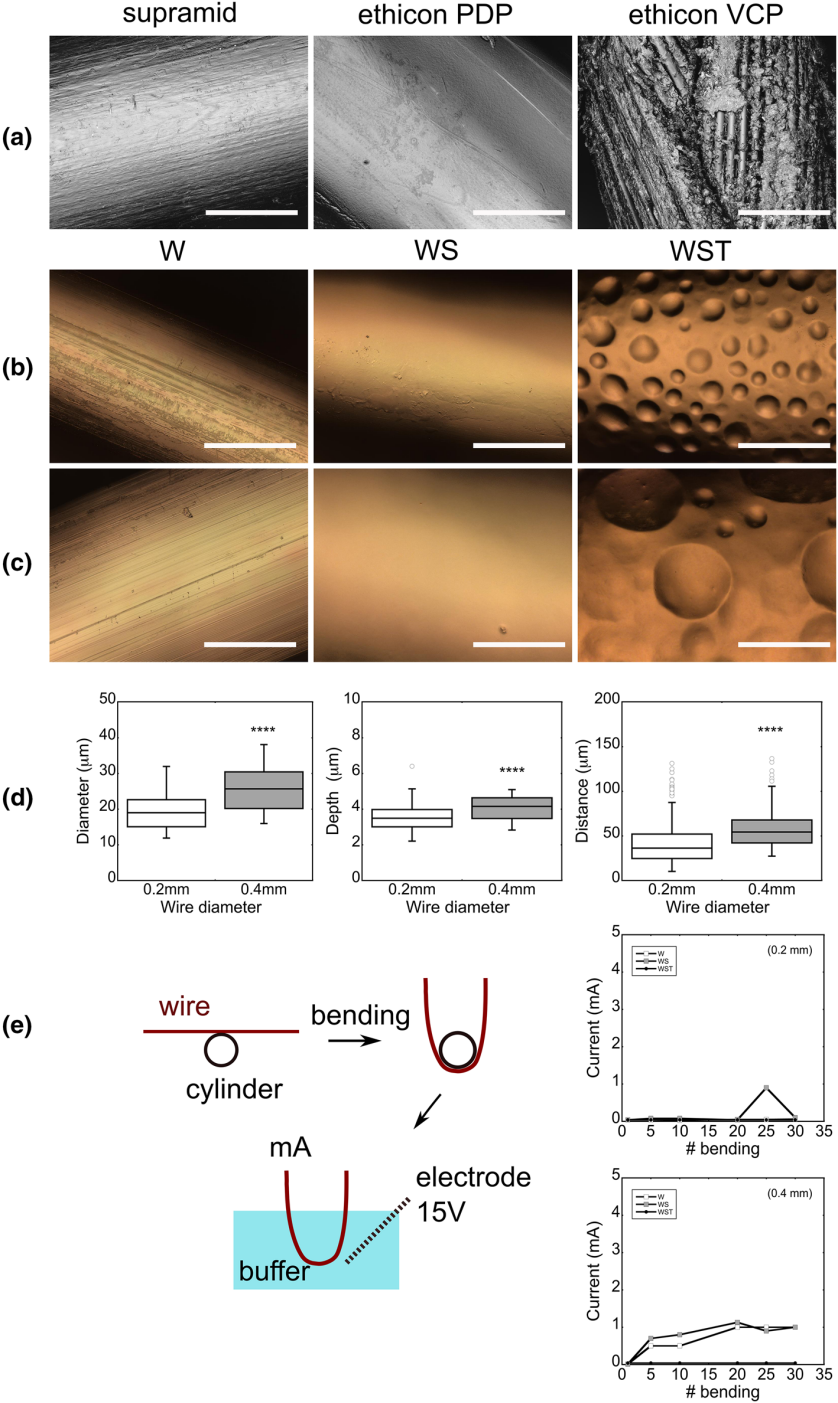
Functionalization of thin conductive wires with selected physical characteristics. (**a**) Commercially available surgical sutures are eminent examples of percutaneous sub-millimeter objects with relatively biocompatible properties. From left to right: monofilament supramid (0.4 mm) and ethicon PDP 305 (0.3 mm) sutures. Ethicon VCP 215 (0.3 mm) is made of absorbable vicryl with distinct braided architecture for enhanced adhesion on the contacting tissue. Enameled conductive wires (W) with a diameter of (**b**) 0.2 mm and (**c**) 0.4 mm. Chemical modification with a silicone layer (WS). Physical modification with breath features (WST). (**d**) Diameter, depth, and inter-space distance of breath features in WST wires were captured by specialized confocal microscopy (von Petersdorff-Campen et al. 2021) and quantified by a custom-made MATLAB protocol (Wu et al. 2021). (**e**) Scheme of the electromechanical testing protocol for monitoring the insulation damage in response to the deformation of W, WS, and WST wires. *P* values indicated for *P* < 0.05 (*), *P* < 0.01 (**), *P* < 0.001 (***), and *P* < 0.0001 (****). Scale bars are 100 μm

The different limitations associated with the use of drivelines in LVADs urge the design of new power transfer systems to support IMDs without the problem of DLIs. A potential prototype to limit DLIs as well as FBR shall involve the use of skin substitutes with individually incorporated metallic wires (Fig. 3e). The novelty of our proposed solution is centered around the righteous selection and modification of the size and surface characteristics of conductive wires (i.e., material and topography). In particular, the proposed prototype aims to leverage the ability of the skin tissue to more effectively seal and heal around small size objects. Despite the limited amount of data on how the size of drivelines affects the incidence of DLIs (Imamura et al. 2017), small percutaneous objects have lower contact area with the abdominal wall limiting the progress of biofilm migration. In a similar context, thin, sub-millimeter sutures repair skin incisions without significant wound formation (Fig. 4a), while needles and implants of smaller diameter are also shown to reduce the underlying trauma and FBR (Helton et al. 2011). Driven by this evidence, we chose enameled copper wires with significantly smaller diameter (*D* = 0.2 and *D* = 0.4 mm, Distrelec) compared to LVAD drivelines (*D* = 6 mm in HeartMate 3) as the main conductive part of our prototype (Fig. 3e). To the extent of our knowledge, this is the first time that a similar configuration of thin conductive wires is proposed as part of a power transfer system in IMDs, and LVADs in particular.

However, apart from the size, the chemical profile of the implanted objects may also lead to adverse complications, such as FBR (Anderson et al. 2008). Because silicone is a rather common material on the surface of medical devices (Curtis and Steichen 2020), we modified our conductive wires by adding an outer layer of silicone (Fig. 4b, c). Yet, silicone surfaces are vulnerable to fibrotic responses (Curtis and Steichen 2020). In effect of that, previous studies modified silicone surfaces with rational topographical details to regulate cell adhesion against fibrosis (Park et al. 2019; Robotti et al. 2018). Motivated by that, we pursued to obtain control over the surface topography of the conductive wires which due to their 3D geometry are not compatible to conventional micropatterning techniques (Quist and Oscarsson 2010). In return, we developed an innovative method of free-form topography to successfully introduce breath features by the condensation of water droplets on top of semi-cured silicone substrates (Fig. 4b, c) (Wu et al. 2021). In contrast with other methods for imprinting breath topography (Kawano et al. 2013; Martínez-Campos et al. 2016), our strategy employs a solvent-free approach that obtains spatial profiles of different features (i.e., depth, diameter, and inter-space distance) on 0.2 mm and 0.4 mm wires, respectively (Fig. 4d).

**Fig. 5:**
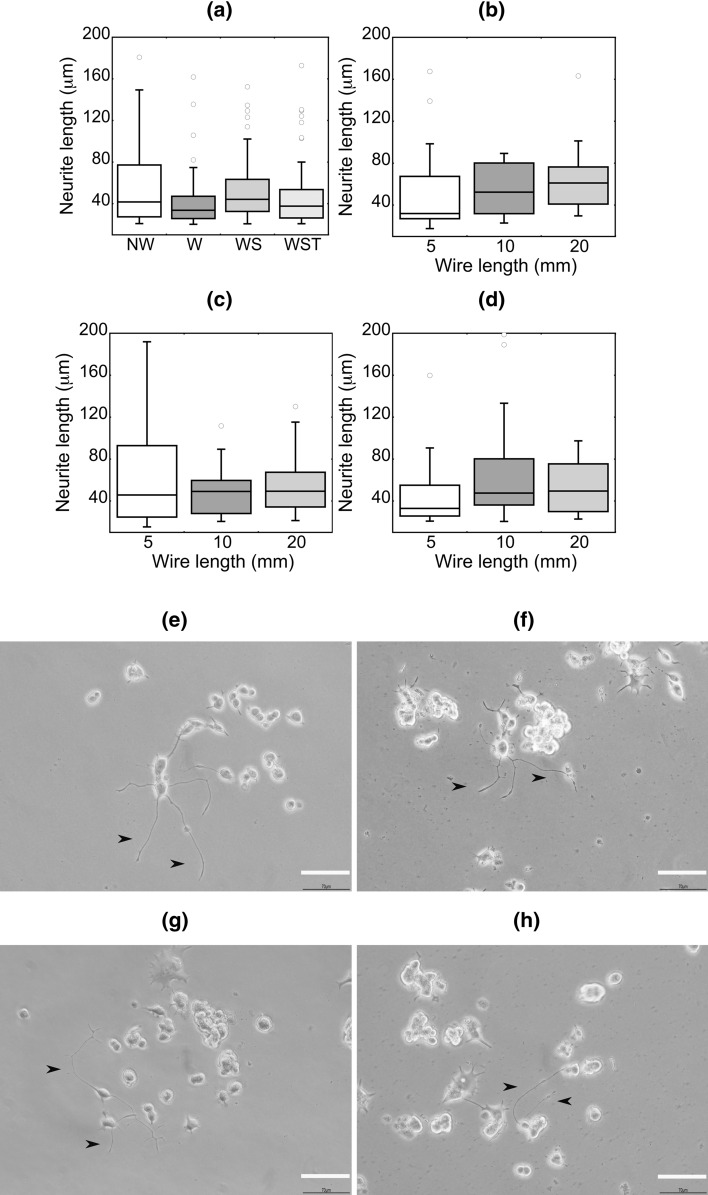
Enamel copper wires induce non-cytotoxic effects in in vitro culture. (**a**–**d**) Box-&-Whisker plots for the neurite length (> 20 μm) grown by PC 12 cells during co-culture with different wire conditions. For all the conditions *P* value > 0.05. (**e**,**f**) Representative pictures of neurites in PC 12 cells (black arrows). *P* values indicated for *P* < 0.05 (*), *P* < 0.01 (**), *P* < 0.001 (***), and *P* < 0.0001 (****). Scale bars are 70 μm

Furthermore, the small diameter of the metallic wires shall strongly reduce their flexural stiffness so to effectively protect the adjacent skin from external forces. On the other hand, lower stiffness entails the risk of larger mechanical movements by the LVAD drivelines at the exit site and the occasional destruction of their electrical insulation (Coyle et al. 2020). For this reason, we developed a customized protocol to assess the electrical resistance of the wires as a function of controllable mechanical deformation (Fig. 4e). In this test, the electrical current conducted from the buffer to the enameled wires shall be indicative of the underlying insulation defects. Since the conducted current is lower than previously reported levels with hazardous effects on the human body (Fish and Geddes 2009), it suggests that the small diameter wires are eligible for further investigation for the generation of new power transfer systems. In this direction, we also explored the biocompatibility of the wires through an established cytotoxicity assay, according which neurite growth of rat adrenal phaeochromocytoma cells (PC 12) correlates with cell apoptosis in vitro (Bernardi et al. 2017; Ferrari et al. 2010). Our findings demonstrate negligible changes on the length of neurites, suggesting the non-cytotoxic effects of the investigated wires (Fig. 5). Beyond the scope of this review, future studies shall proceed to a thorough characterization of the electromechanical and biological properties of the wires under environmental conditions that resemble those of implanted LVAD drivelines.

## Future outlook

The design of new models of conductive skin is expected to grow along with the need for more advanced clinical protocols against DLIs. Apart from offering a potential solution to power LVADs, the design of new systems of conductive skin may expand the capabilities of other life-supporting technologies, such as wearable artificial organs (Gura et al. 2016; Lu et al. 2020). Since infection and FBR are major complications related with IMDs, the functional characteristics of conductive skin shall work against these problems. In this direction, the progress of mechanobiology and tissue engineering provides a guide to engineer systems of conductive skin with minimal tissue morbidity. Specific experimental efforts shall proceed with the formulation of 3D skin substitutes that contain surface-functionalized conductive wires (Fig. 3e) with the capacity to power IMDs without severe complications. The pre-clinical evaluation shall attest the effects of different conductive skin systems in host organisms that are suitable for exploratory LVAD studies. In addition, the use of dynamic bioreactors offers a powerful in vitro strategy to assess the performance of new systems of conductive skin under non-static conditions with controllable mechanical and biological signals (Wahlsten et al. 2021). Overall, the mechanobiology-directed design of conductive skin, in synergy with the development of complementary health technologies, has the potential to revolutionize the capabilities of IMDs and improve patient management against chronic diseases.
